# Identification of the C-Terminal GH5 Domain from *Cb*Cel9B/Man5A as the First Glycoside Hydrolase with Thermal Activation Property from a Multimodular Bifunctional Enzyme

**DOI:** 10.1371/journal.pone.0156802

**Published:** 2016-06-03

**Authors:** Rong Wang, Li Gong, Xianli Xue, Xing Qin, Rui Ma, Huiying Luo, Yongjie Zhang, Bin Yao, Xiaoyun Su

**Affiliations:** 1 Key Laboratory for Feed Biotechnology of the Ministry of Agriculture, Feed Research Institute, Chinese Academy of Agricultural Sciences, Beijing, 100081, P. R. China; 2 School of Life Sciences, Shanxi University, Taiyuan, Shanxi, 030006, P. R. China; 3 College of Animal Sciences, Zhejiang University, Hangzhou, 310058, P. R. China; Weizmann Institute of Science, ISRAEL

## Abstract

*Caldicellulosiruptor bescii* encodes at least six unique multimodular glycoside hydrolases crucial for plant cell wall polysaccharides degradation, with each having two catalytic domains separated by two to three carbohydrate binding modules. Among the six enzymes, three have one N- or C-terminal GH5 domain with identical amino acid sequences. Despite a few reports on some of these multimodular enzymes, little is known about how the conserved GH5 domains behave, which are believed to be important due to the gene duplication. We thus cloned a representative GH5 domain from the C-terminus of a multimodular protein, i.e. the bifunctional cellulase/mannanase *Cb*Cel9B/Man5A which has been reported, and expressed it in *Escherichia coli*. Without any appending CBMs, the recombinant *Cb*Man5A was still able to hydrolyze a variety of mannan substrates with different backbone linkages or side-chain decorations. While *Cb*Man5A displayed the same pH optimum as *Cb*Cel9B/Man5A, it had an increased optimal temperature (90°C) and moreover, was activated by heating at 70°C and 80°C, a property not ever reported for the full-length protein. The turnover numbers of *Cb*Man5A on mannan substrates were, however, lower than those of *Cb*Cel9B/Man5A. These data suggested that evolution of *Cb*Man5A and the other domains into a single polypeptide is not a simple assembly; rather, the behavior of one module may be affected by the other ones in the full-length enzyme. The differential scanning calorimetry analysis further indicated that heating *Cb*Man5A was not a simple transition state process. To the best knowledge of the authors, *Cb*Man5A is the first glycoside hydrolase with thermal activation property identified from a multimodular bifunctional enzyme.

## Introduction

The thermophilic bacterium *Caldicellulosiruptor bescii* (previously classified as *Anaerocellum thermophilum*) optimally growing at 75°C is distinguished by its excellent capacity to degrade crystalline cellulose [1]. It is also able to acquire energy from hemicellulose including xylan and mannan and even untreated switchgrass and poplar [1]. These indicate that the microbe has an array of robust, thermophilic glycoside hydrolases that can efficiently deconstruct plant cell wall polysaccharides (PCWP) into simple sugars. Many endeavors have been carried out in the characterization of the cellulase and xylan-degrading enzymes of *C*. *bescii* [2,3,4,5].

**Fig 1:**
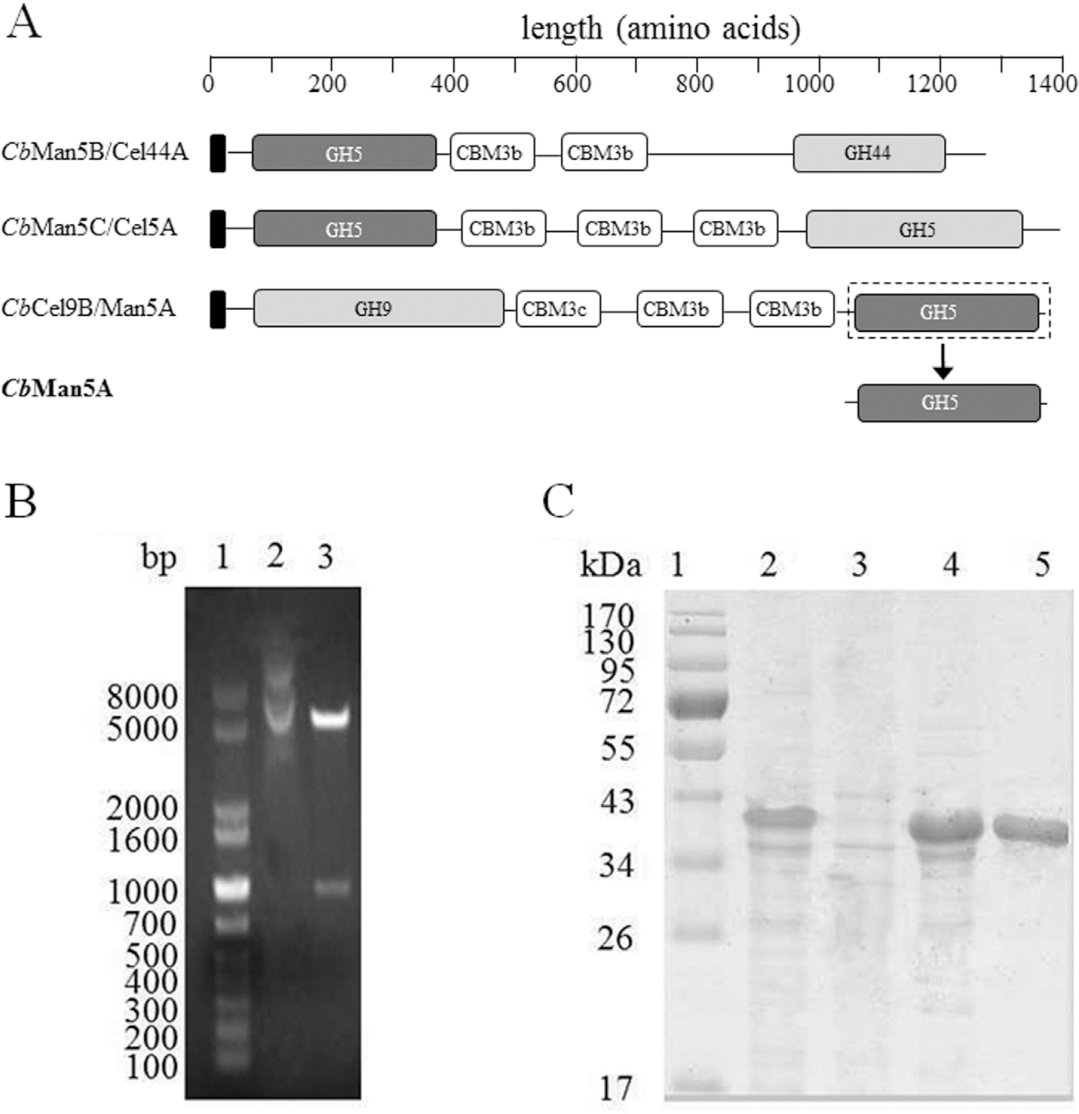
Cloning, expression, and purification of *Cb*Man5A. A: Domain structure showing the three unique multimodular proteins with a conserved GH5 domain. The conserved GH5 domains are filled with dark grey, the signal peptides are filled with black, and the other domains are filled with white (CBMs) or light grey (catalytic domains). *Cb*Man5B/Cel44A, *Cb*Man5C/Cel5A, and *Cb*Cel9B/Man5A are also referred as Athe_1859, Athe_1866, and Athe_1865, respectively. B: Restriction digestion of pET-cbMan5A. Lane 1: DNA molecular mass marker; 2: undigested pET-cbMan5A; 3: pET-cbMan5A digested with *Eco*RI and *Not*I. C: SDS-PAGE analysis of purified *Cb*Man5A. Lane 1: protein molecular mass marker; 2: whole cell lysate of pET-cbMan5A/BL21(DE3); 3: cell debris; 4: supernatant; 5: purified *Cb*Man5A.

One striking finding for the PCWP-degrading glycoside hydrolases of *C*. *bescii* is the existence of a large number of multimodular enzymes. The genome of *C*. *bescii* encodes at least six unique multimodular glycoside hydrolases bearing two catalytic domains separated by two to three carbohydrate binding modules (CBMs) [2]. Notably, these multimodular enzymes are encoded by genes within a single but crucial PCWP-utilization gene cluster [6,7]. Such a special domain organization in a single large polypeptide appears to represent a novel naturally occurring paradigm of glycoside hydrolases for hydrolysis of PCWP. The high efficiency of CelA (or *Cb*Cel9A/Cel48A [8]), a cellulase with one N-terminal GH9 endoglucanase and a C-terminal GH48 exoglucanase, has been ascribed to this special domain arrangement [4]. Interestingly, among the six multimodular glycoside hydrolases, three have one N- or C-terminal GH5 domain (Fig 1A) with the same amino acid sequence (S1 Fig). Despite a few reports on some of these multimodular enzymes, little is known about the biochemical behavior of the conserved GH5 domains, which are believed to be important due to the gene duplication.

We thus cloned a representative GH5 domain from the C-terminus of a multimodular protein, i.e. the bifunctional cellulase/mannanase *Cb*Cel9B/Man5A which has been reported [2], and expressed it in *Escherichia coli*. Biochemical analyses were carried out on the purified protein.

## Results

### Gene Cloning, Expression, and Purification of *Cb*Man5A

Three multimodular enzymes were identified to bear one identical GH5 domain existing at either the N- (*Cb*Man5B/Cel44A, or Athe_1859, GenBank accession number: ACM60947; *Cb*Man5C/Cel5A, or Athe_1866, ACM60954) or C-terminus (*Cb*Cel9B/Man5A, or Athe_1865, ACM60953) (Fig 1A). The gene coding for the C-terminal GH5 domain of *Cb*Cel9B/Man5A was ligated into pET-28a(+) to obtain pET-CbMan5A. The insertion of *cbMan5A* was verified by restriction digestion of the recombinant plasmid using *Eco*RI and *Not*I (Fig 1B) and DNA sequencing (data not shown). The recombinant *Cb*Man5A expressed in *E*. *coli* was purified by immobilized metal affinity chromatography (IMAC) as analyzed by SDS-PAGE (Fig 1C).

### Substrate Specificity of *Cb*Man5A

**Fig 2:**
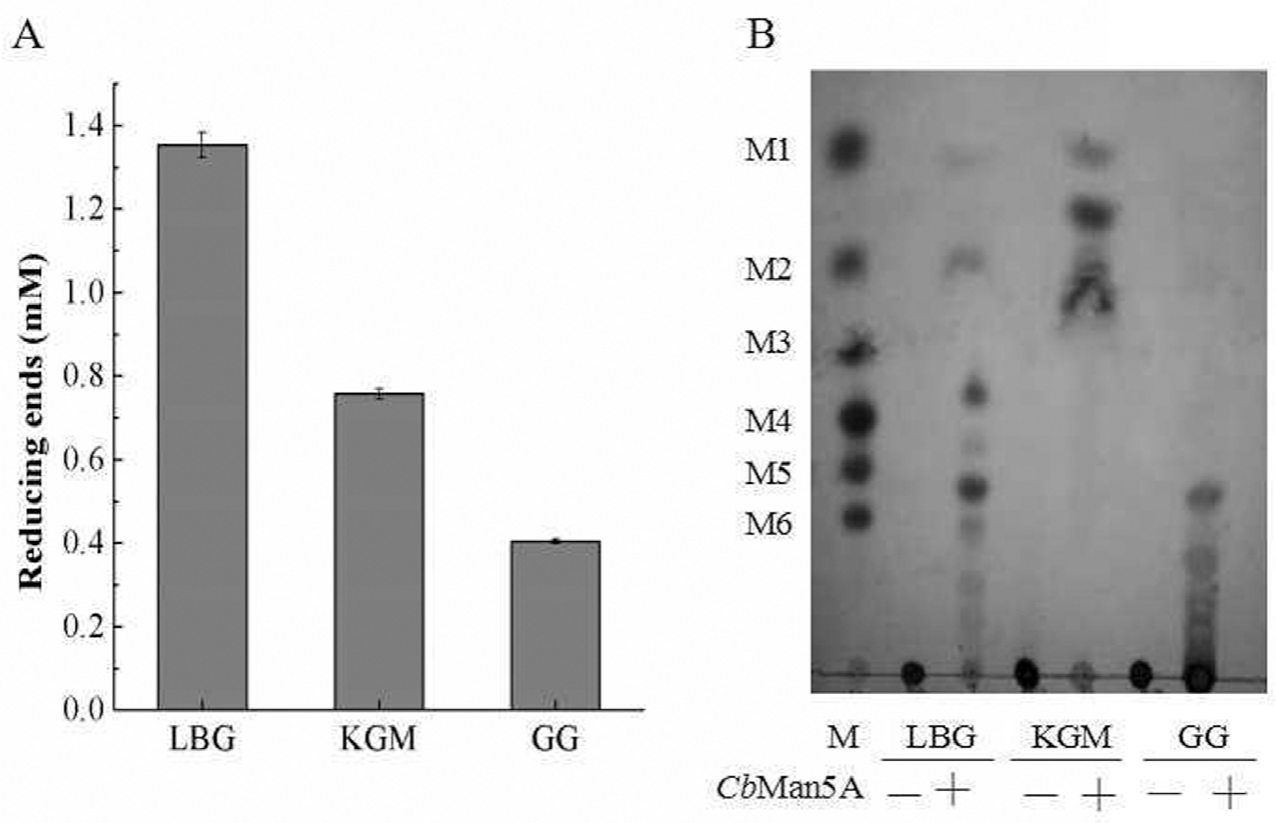
Hydrolysis of mannan substrates. A: Substrate specificity of *Cb*Man5A on mannans. Although mannan, cellulose, and xylan substrates were included in the screening, only the mannan substrates with significant signal of reducing sugars in the 15-min assay were shown here. B: Hydrolysis products of *Cb*Man5A on various mannans as analyzed by thin-layer chromatography. M1: mannose; M2-M6: mannobiose to mannohexaose. LBG: locust bean gum; KGM: konjac glucomannan; GG: guar gum.

*Cb*Man5A was most active in degrading the galactomannan locust bean gum (LBG), followed by konjac glucomannan (KGM, a polysaccharide with a mixed glucose-mannose linkage) and another galactomannan guar gum (GG) (Fig 2A), demonstrating that it could hydrolyze a variety of mannan substrates. However, no reducing sugars were released from cellulose and xylan in the short time (15 min) assay (Fig 2A) unless the incubation was prolonged to 12 h (data not shown). From LBG, *Cb*Man5A released mannose, mannobiose, and mannooligosaccharides with higher degrees of polymerization or side chain modifications as end products (Fig 2B). For GG, the major products were a mannooligosaccharide appearing on the TLC plate between mannopentaose and mannohexaose and those with higher degrees of polymerization (Fig 2B). In contrast, *Cb*Man5A released mannose and oligosaccharides with low degrees of polymerization from KGM (Fig 2B).

### Effects of pH and Temperature on the Activity and Stability of *Cb*Man5A

**Fig 3:**
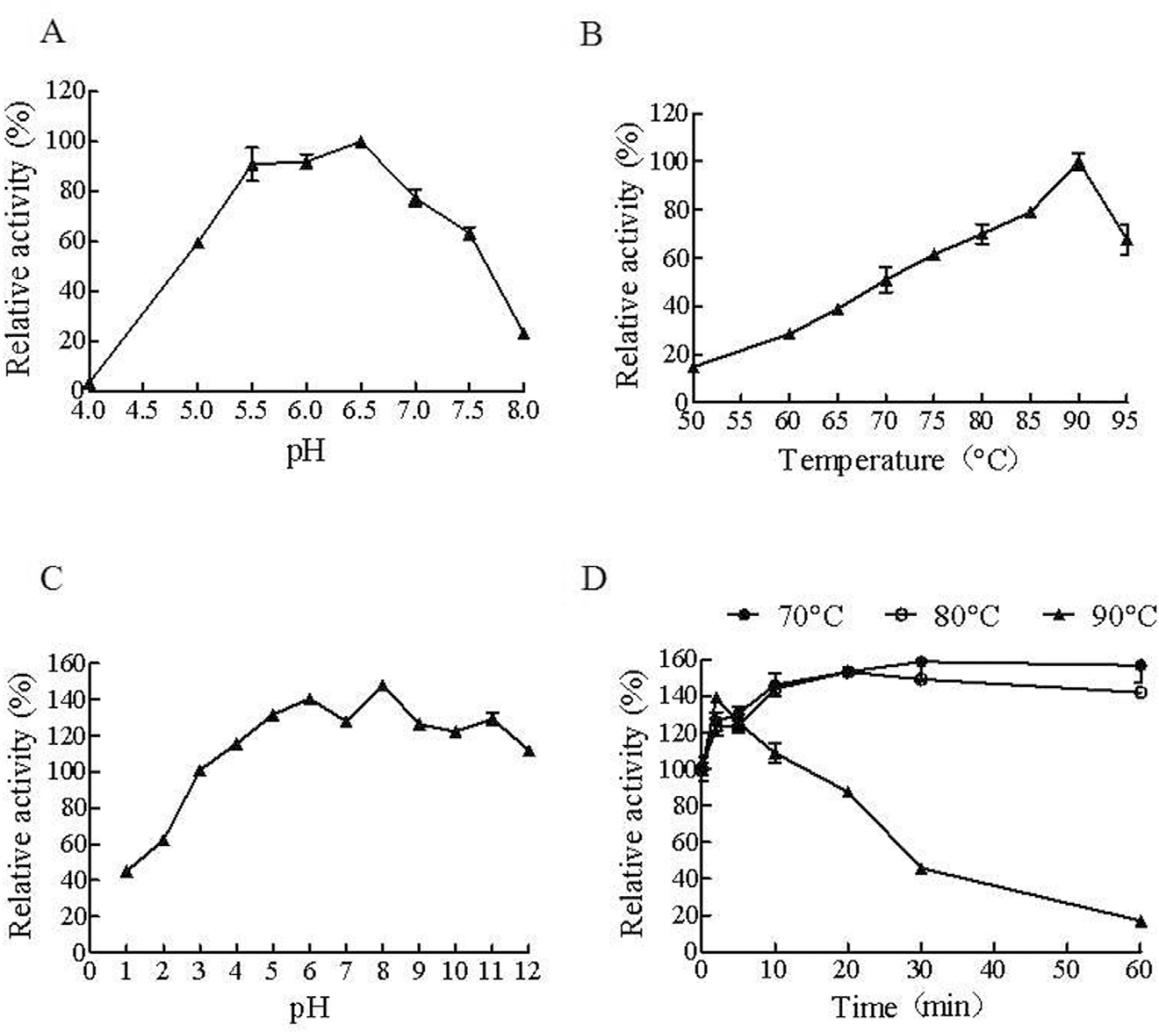
Effects of pH and temperature on the activity and stability of *Cb*Man5A. A: pH optimum of *Cb*Man5A. B: Temperature optimum of *Cb*Man5A. C: Stability of *Cb*Man5A at various pHs. The enzyme was pretreated at a given pH for 1 h before activity measurement. D: Thermotolerance of *Cb*Man5A. The activities of the *Cb*Man5A under different conditions or treatments were represented as relative activity (in percentage) by dividing the activities against the reference activity, which was the maximal activity under a certain situation (for A, pH6.5: 1,713 U/mg; for B, 90°C: 1,566 U/mg) or the activity before treatment (for C, 1,482 U/mg; for D, 976 U/mg, 70°C; 1,082 U/mg, 80°C; 1,005 U/mg, 90°C).

*Cb*Man5A had an optimal pH of 6.5 (Fig 3A) identical to that of *Cb*Cel9B/Man5A. The optimal temperature of *Cb*Man5A was 90°C, higher than that of *Cb*Cel9B/Man5A (85°C). At the temperatures from 75–95°C, the enzyme had relative activities of above 60% (Fig 3B). While pretreatment at pH1.0 and 2.0 decreased the activity of *Cb*Man5A substantially, incubation at pH3.0 for 1 h appeared not to affect its activity, and pH4.0 to pH12.0 tended to increase the activity significantly (Fig 3C). At 90°C, *Cb*Man5A quickly lost its activity to 17% after 60 min (Fig 3D). However, no loss of activity was observed for incubation at 70°C and 80°C, indicating that *Cb*Man5A has a sound thermostability. Interestingly, significant increases of residual activity (up to 159% and 153%) were observed for *Cb*Man5A pretreated at both 70°C and 80°C after 1 h (Fig 3D).

### Kinetic Analysis of *Cb*Man5A

For LBG, KGM, and GG, the specific activities of *Cb*Man5A were 1,691, 690, and 505 U/mg, respectively, while those for Avicel, filter paper, CMC-Na, and beechwod xylan were much lower (Table 1). *Cb*Man5A had a *k*_cat_ of 1,043 and 514 s^-1^, *K*_*m*_ of 1.7 and 0.9 mg ml^-1^, and catalytic efficiency of 602.9 and 571.1 s^-1^ ml mg^-1^ on LBG and KGM, respectively. The kinetic parameters of *Cb*Man5A on GG were not obtained since the reaction could not be saturated even at very high concentration of the substrate. Compared with other mannnases, *Cb*Man5A is a robust mannanase degrading galactomannan (Table 1).

**Table 1:** Biochemical properties of *Cb*Man5A in comparison with other mannanases.

Enzyme	Organism	Optimal pH	Optimal temperature(°C)	Specific activity (unit, substrate)	*k*_*cat*_ (s^-1^)	*K*_*m*_ (mg ml^-1^)	Reference
*Cb*Man5A	*C*. *bescii*	6.5	90	1,691 (U/mg, LBG)	1043	1.7	This.work
				690 (U/mg, KGM)	514	0.9	This.work
				505 (U/mg, GG)	–	–	This.work
				1.4 (mU/mg, Avicel)	–	–	This.work
				0.5 (mU/mg, Filter paper)	–	–	This.work
				2.2 (mU/mg, CMC-Na)	–	–	This.work
				5.9 (mU/mg, Beechwood xylan)	–	–	This.work
Man5A1	*Talaromyces leycettanus*	4.5	90	2,160 (U/mg, LBG)	–	1.9	[19]
Man5A2	*Talaromyces leycettanus*	4.0	85–90	1,800 (U/mg, LBG)	–	2.2	[19]
Man5XZ3	*Aspergillus nidulans*	5.0	80	186 (U/mg, LBG)	–	0.9	[20]
ManBK	*Aspergillus niger*	4.5	80	–	292	2.2	[21]
MAN-P	*Aspergillus niger*	4.5	80	3,049 (U/ml, LBG)	–	–	[22]
rMan5P1	*Neosartorya fischeri*	4.0	80	1,703 (U/mg, LBG)	–	0.8	[23]
Man5XZ7	*Thielavia arenaria*	5.0	75	–	–	5.3	[24]
Man5A	*Humicola insolens*	5.5	70	1,122 (U/mg, LBG)	–	1.5	[25]
AaManA	*Alicyclobacillus acidocaldarius*	5.5	65	–	340	2.4	[26]
ManA	*Bacillus subtilis*	7.0	60	415.2 (U/ml, LBG)	–	–	[27]
MANN	*Aspergillus sulphureus*	2.4	50	366 (U/mg, LBG)	–	0.9	[28]
ManA	*Xanthomonas campestris*	7.0	37	282.55 (U/mg, LBG)	–	0.88	[29]

### Effect of Chemicals and Metal Ions on the Activity of *Cb*Man5A

The presence of Co^2+^, K^+^, Na^+^, Ni^2+^, Mn^2+^, and Zn^2+^, and surprisingly β-mercaptoethanol, enhanced its activity by over 10% (S1 Table). Only Ag^+^ could strongly inhibit the enzyme by repressing the activity of *Cb*Man5A to 9.0%.

### Thermal Unfolding of *Cb*Man5A Fitted Well to a Non-2 State Model

**Fig 4:**
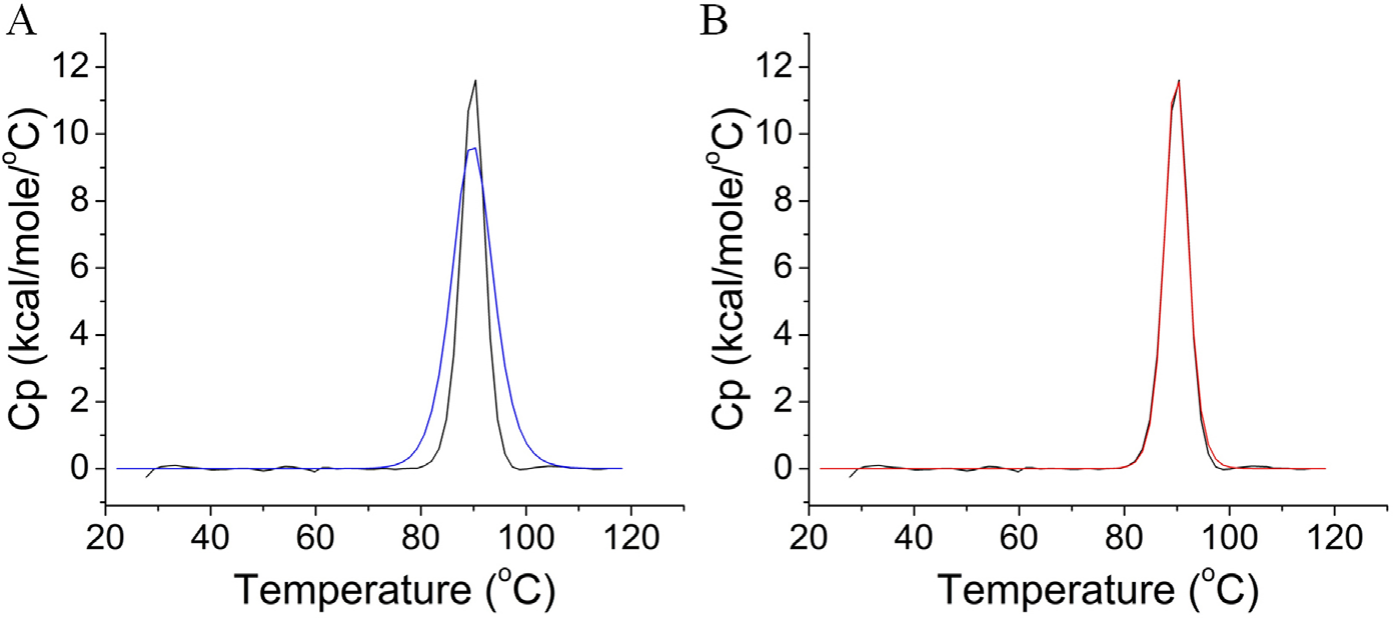
Heating of *Cb*Man5A as analyzed by DSC. A and B: Fitting of the DSC data to either a simple 2-state (A) or a non-2-state model (B). The black solid lines in A and B are the raw DSC data, while the blue and red solid lines in A and B are the fitted curves with the 2-state or non-2-state model, respectively.

Fitting of the raw DSC data according to a simple 2-state model generated poor overlay of the two curves, as illustrated in Fig 4A. However, the DSC data fit much better to a non-2 state model with one transition stage (Fig 4B). The T_m_ (transition midpoint) was 89.9°C, with a *Δ*H of 6.903×10^4^ cal/mol and *Δ*H_v_ of 1.812×10^5^ cal/mol, respectively.

## Discussion

The multiple GH5 endo-mannanase orthologs are thought to cooperate with a GH2 β-mannosidase in degrading mannans into mannose for metabolism by *C*. *bescii* [9]. With the optimal temperature of 90°C, *Cb*Man5A is one of the most thermophilic mannanases that have ever been characterized (Table 1). *Cb*Man5A is a robust endo-mannanase hydrolyzing a variety of mannan substrates and resistant to many metals and chemicals, indicating that it may serve as a good candidate enzyme with application potential in industry. Noticeably, heating *Cb*Man5A at 70°C and 80°C for 1 h improved, rather than diminished, its enzymatic activity. In line with this, the DSC analysis indicated that heating *Cb*Man5A is not a simple unfolding process with only two states. Thermal activation of enzymes is only discovered for a few enzymes such as an apple polyphenol oxidase [10], a glutamate dehydrogenase from *Pyrococcus furiosus* [11], and an alkaline lipase from *Pseudomonas* sp. [12]. The rare examples also include glycoside hydrolases such as an endoglucanase from *Thermomonospora curvata* [13] and a β-galactosidase from *Thermotoga naphthophila* [14]. To the best knowledge of the authors, *Cb*Man5A is the first glycoside hydrolase with thermal activation property derived from a multimodular bifunctional enzyme.

Without any CBM3 modules, *Cb*Man5A is still able to degrade a variety of mannans as *Cb*Cel9B/Man5A does, indicating that the CBM3s are not the primary determinant of the substrate specificity of *Cb*Man5A for these model substrates. However, the biochemical properties of *Cb*Man5A apparently differed from those of the full-length enzyme *Cb*Cel9B/Man5A. The *K*_*m*_ of *Cb*Man5A on LBG (1.7 mg/ml) was higher, while that on KGM (0.9 mg/ml) was lower, than those of *Cb*Cel9B/Man5A (0.62 and 1.84 mg/ml, respectively). The *k*_cat_s of *Cb*Man5A on LBG and KGM were 1,043 and 514 s^-1^, respectively, both lower than those of *Cb*Cel9B/Man5A (1,420 s^-1^ and 1,068 s^-1^, respectively). Notably, the thermal activation property is not observed for the full-length enzyme *Cb*Cel9B/Man5A (S2 Fig and [2]). Therefore, the data suggested that evolution of *Cb*Man5A and the other domains (one GH9 and three CBM3s) into a single polypeptide is not a simple assembly since the missing of the N-terminal CBMs and GH9 catalytic module has complex effects on the enzyme characters of *Cb*Man5A. Our finding further supports the hypothesis that the catalytic and accessory modules in the multi-domain glycoside hydrolases may not be simply tandem-linked but rather functionally coupled [2,15].

The recurring of GH5 mannanases is not the only case of gene duplication in multimodular glycoside hydrolases in *C*. *bescii*. The rest three among the six multimodular enzymes (CelA, GenBank accession number: ACM60955; *Cb*Xyn10C/Cel48B, ACM60945; and Athe_1860, ACM60948) all have an identical C-terminal GH48 cellobiohydrolase [16]. The recruiting of *Cb*Man5A into the multimodular *Cb*Cel9B/Man5A apparently enhanced its activity in terms of the turnover numbers (*k*_cat_); however, the thermal activation character, a property that would be more favorable for adaptation to high temperature environment, is not retained. Since there is no evidence of significant cleavage of *Cb*Man5A from the full-length enzyme *Cb*Cel9B/Man5A [4], the heat activation property of *Cb*Man5A is an interesting phenomenon but may be non-biologically relevant. Note, in contrast, there is significant cleavage of GH48 modules from the multimodular cellulases [4]. Therefore, it may be interesting to explore how differently the GH48 module would behave either as a single domain protein or in the context of a multimodular protein. It may also be inferred that, the thermodynamics effect in addition to the enzymology of associated domains has to be considered when one designs artificial multimodular bifuntional glycoside hydrolases.

In conclusion, we identified the C-terminal GH5 mannanase of *Cb*Cel9B/Man5A as the first glycoside hydrolase with heat activation property from a multimodular bifunctional enzyme. *Cb*Man5A is a highly thermophilic, robust endo-mannanase hydrolyzing a variety of mannans, potentiating it as a candidate for industry application. *Cb*Man5A displays similar but still differing characteristics to those of the full-length *Cb*Cel9B/Man5A, suggesting that the multimodular and bifunctional enzymes are more than a simple assembly of several independent domains; rather, the behavior of one module may be affected by the other ones in the full-length enzyme.

## Materials and Methods

### Plasmid Construction

The gene encoding the C-terminal GH5 (aa 1048 to 1360, designated *Cb*Man5A) of *Cb*Cel9B/Man5A was amplified from the genomic DNA of *C*. *bescii* DSM 6725 (DSMZ, Braunschweig, Germany) with the primer pairs of Pman5A_F (5’-GGCGAATTCCAGGAGCCGAGTGGAGCGACACCAAC-3’, underlined for *Eco*RI) and Pman5A_R (5’-ATGGGCGGCCGCTTATTCAGCACcaatcgcattag-3’, underlined for *Not*I) using the PrimeSTAR HS DNA Polymerase (TaKaRa, Dalian, China). The PCR products were digested with *Eco*RI and *Not*I, and then ligated into the pET-28a(+) plasmid (Merck, Darmstadt, Germany) to obtain pET-cbMan5A.

### Gene Expression and Protein Purification

The pET-cbMan5A plasmid was transformed into BL21(DE3) (Transgen, Beijing, China) chemically competent cells. Five to six colonies were inoculated into 200 ml of LB medium and the culture was shaken at 37°C. When the OD_600_ of the culture reached 0.6, IPTG was added to the culture at a final concentration of 0.2 mM, and the culture was continued for 16 h. The *E*. *coli* cells were harvested by centrifugation and re-suspended in a binding buffer containing 20 mM Tris-HCl, 500 mM NaCl, pH7.6. The cytosol containing the recombinant *Cb*Man5A was released by sonication. *Cb*Man5A was purified by using immobilized-metal affinity chromatography (IMAC) with an column-equilibration buffer of NTA0 containing 50 mM Tris-HCl, 500 mM NaCl, pH7.5 and elution buffers with the NTA0 containing a series of step gradient concentrations of imidazole (40 to 400 mM).

### Determination of the Substrate Specificity of *Cb*Man5A

All polysaccharides, unless otherwise mentioned, were purchased from Sigma-Aldrich (St. Louis, MO). Ten μM of *Cb*Man5A was incubated in a McIlvaine buffer containing 200 mM sodium phosphate, 100 mM sodium citrate, pH 6.0 with a series of plant cell wall polysaccharides including 5 mg/ml of locust bean gum (LBG), guar gum (GG), and konjac glucomannan (KGM, from Megazyme, Wicklow, Ireland), and 10 mg/ml of beechwood xylan, sodium carboxymethyl cellulose (CMC-Na), Avicel, and filter paper. The reaction was carried out at 70°C for 15 min. For the last four polysaccharides, a prolonged reaction was also carried out at 70°C for 12 h. The released reducing sugars were determined using the 3,5-dinitrosalicylic acid (DNS)method [17].

### Thin-Layer Chromatorgraphy (TLC)

The TLC method described by Moon et al. [18] was employed to analyze the hydrolysis products of *Cb*Man5A on different polysaccharides. Two nM of *Cb*Man5A were incubated with 2.5 mg/ml of LBG, KGM, and GG in 5 mM McIlvaine buffer (pH 6.5) in a total volume of 500 μl at 80°C for 12 h. Appropriate amounts of the hydrolysates were applied onto a silica gel (Silica gel 60 F254, Merck, Darmstadt, Germany) and air dried. The plate was developed using a solution containing n-butanol-acetic acid-H_2_O with a volumetric ratio of 10:5:1. The plate was air dried, sprayed with a solution containing H_2_SO_4_-ethanol with a volumetric ration of 1:19, and heated at 110°C for 10 min until the products could be clearly visualized.

### Effect of pH and Temperature on the Activity and Stability of *Cb*Man5A

For optimal pH determination, 0.2 μM *Cb*Man5A was incubated with 5 mg/ml of LBG in the McIlvaine buffers with different pHs (pH4.0–8.0) at 70°C for 10 min. For optimal temperature, *Cb*Man5A was incubated with 5 mg/ml of LBG in the McIlvaine buffers at pH6.5 at temperatures ranging from 50°C to 90°C with a gradient of 5°C. The released reducing sugars were determined using the DNS method. To determine the pH-stability, 0.2 μM *Cb*Man5A was first pretreated in the buffers with different pHs (pH1.0–2.0: 100 mM glycine-HCl; pH 3.0–8.0: 100mM McIlvaine buffer; pH 9.0–12.0: 100 mM glycine-NaOH) at 37°C for 1 h. To determine the thermostability, *Cb*Man5A was pre-incubated at 70°C, 80°C, and 90°C for different time periods (2 min, 5 min, 10 min, 20 min, 30 min, and 1 h). Thereafter, for both pH-stability and thermostability experiments, the residual activity was determined at its optimal reaction conditions (pH 6.5 and 90°C). The released reducing sugars were measured using the DNS method. The experiments were repeated for three times.

### Determination of the Enzyme Kinetics

Appropriate amounts of *Cb*Man5A were incubated in the McIlvaine buffer with 0.5 to 5 mg/ml of LBG or KGM at its optimal pH and temperature for 5 min when the released reducing sugars versus time were linear. The *K*_*m*_ and *V*_max_ were calculated according to the Michaelis-Menten equation using the software GraphPad Prism 5.01 (La Jolla, CA). The specific activity was determined by incubating 2 nM of *Cb*Man5A with 5 mg/ml of LBG, GG or KGM at its optimal pH and temperature for 10 min. One unit (U) of specific activity was defined as the amount of enzyme that released one μmol of reducing sugars per minute.

### Effect of Metal Ions and Chemicals on the Enzyme Activity

The salts including NaCl, KCl, CaCl_2_, CoCl_2_, CrCl_3_, NiCl_2_, CuCl_2_, MgCl_2_, FeCl_3_, MnCl_2_, ZnCl_2_, Pb(CH_3_COO)_2_, and AgNO_3_ containing different metal ions, and three chemicals including sodium dodecyl sulfate (SDS), ethylene diamine tetraacetic acid (EDTA), and β-mercaptoethanol, were added with a final concentration of 5 mM into the reaction mixture. The mannanase activity of *Cb*Man5A was measured using 5 mg/ml of LBG under its optimal conditions.

### Differential Scanning Calorimetry (DSC) Analysis

DSC was performed on a Nano-DSC (TA Instruments, New Castle, DE) at a heating rate of 1°C/min and a scanning rate of 1°C/min. The sample (0.2 mg/ml of *Cb*Man5A) were dissolved in 100 mM McIlvaine buffers (pH 7.5).The samples were degassed for 7 min before measurements in a dewar vessel. Then 400 μl of the sample with a 400 μl reference were loaded into the calorimeter cells. As the baseline, the same amount of buffer was measured and then subtracted from measured scan. During all DSC experiments, a constant pressure of 3 atm was maintained to prevent possible degassing of the solution on heating. The test was repeated twice at temperatures between 20°C and 120°C.

## Supporting Information

S1 Fig Amino acid sequence alignment of *Cb*Man5A with the GH5 domains from the other two multimodular proteins of *C*.*bescii*. Athe_1866 and Athe_1859 represent *Cb*Man5C/Cel5A and *Cb*Man5B/Cel44A, respectively.
(DOCX)

S2 Fig Thermostability of the full-length *Cb*Cel9B/Man5A. *Cb*Cel9B/Man5A was incubated at 70°C, 80°C, and 90°C for 1 h. At different time, samples were taken out and measured for the residual mannanase activity. Locust bean gum was used as the substrate. The activities of *Cb*Cel9B/Man5A were represented as relative activity (in percentage) by dividing the activities against the reference activity, which was the activity before treatment.
(DOCX)

S1 Table Effects of metal ions and chemicals on the activity of *Cb*Man5A. (DOCX)
