# Supplementary material for: Identification of the C-Terminal GH5 Domain from CbCel9B/Man5A as the First Glycoside Hydrolase with Thermal Activation Property from a Multimodular Bifunctional Enzyme
Source: PLoS One. 2016 Jun 3;11(6):e0156802. doi: 10.1371/journal.pone.0156802 (PMC4892530; doi:10.1371/journal.pone.0156802)
Supplement: S1 Fig — Athe_1866 and Athe_1859 represent CbMan5C/Cel5A and CbMan5B/Cel44A, respectively. (DOCX) [file pone.0156802.s001.docx]

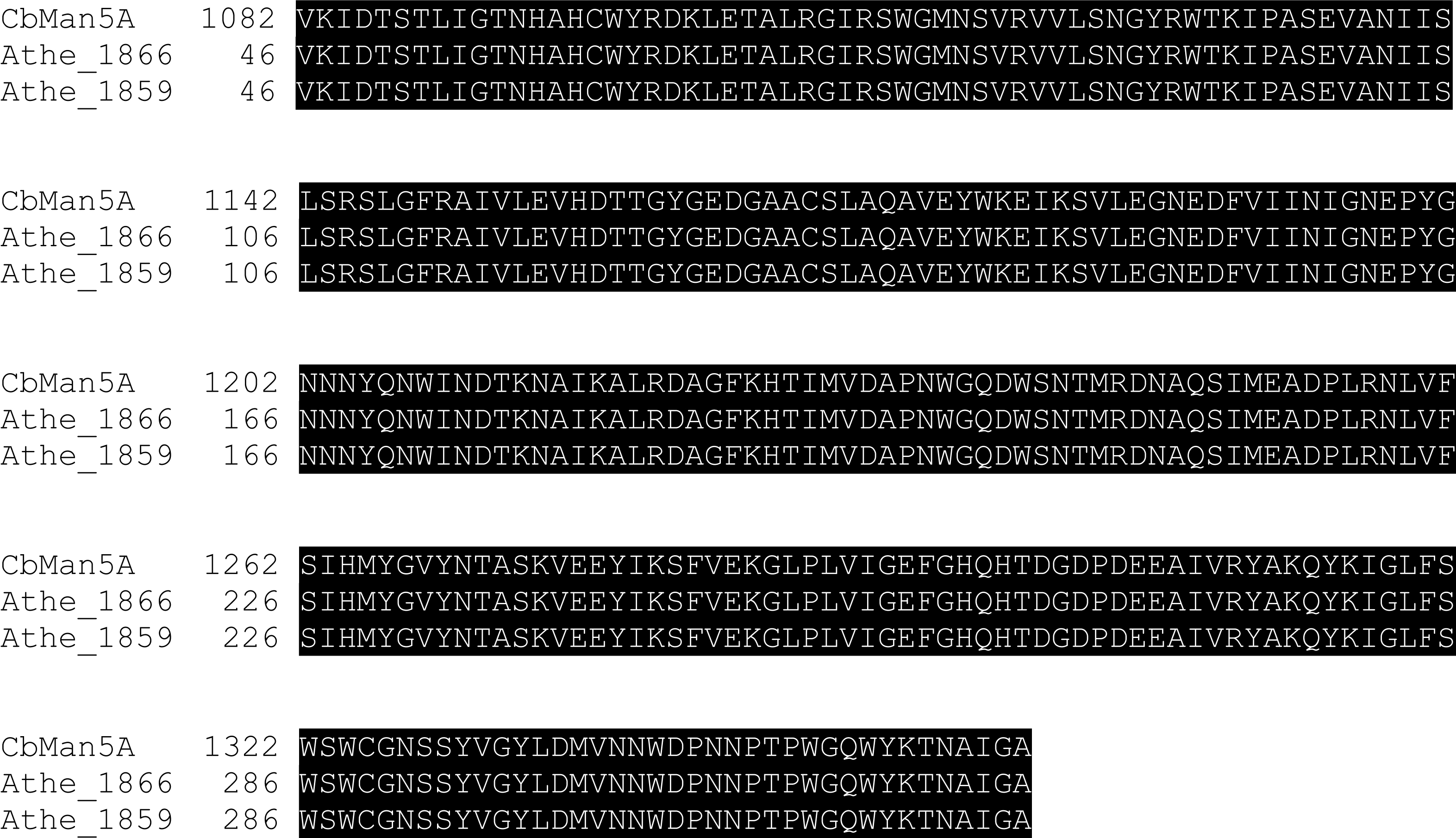


**S1 Fig.. Amino acid sequence alignment of *Cb*Man5A with the GH5 domains from the other two multimodular proteins of *C .bescii.*** Athe_1866 and Athe_1859 represent *Cb*Man5C/Cel5A and *Cb*Man5B/Cel44A, respectively.
