# Supplementary material for: Identification of the C-Terminal GH5 Domain from CbCel9B/Man5A as the First Glycoside Hydrolase with Thermal Activation Property from a Multimodular Bifunctional Enzyme
Source: PLoS One. 2016 Jun 3;11(6):e0156802. doi: 10.1371/journal.pone.0156802 (PMC4892530; doi:10.1371/journal.pone.0156802)
Supplement: S2 Fig — CbCel9B/Man5A was incubated at 70°C, 80°C, and 90°C for 1 h. At different time, samples were taken out and measured for the residual mannanase activity. Locust bean gum was used as the substrate. The activities of CbCel9B/Man5A were represented as relative activity (in percentage) by dividing the activities against the reference activity, which was the activity before treatment. (DOCX) [file pone.0156802.s002.docx]

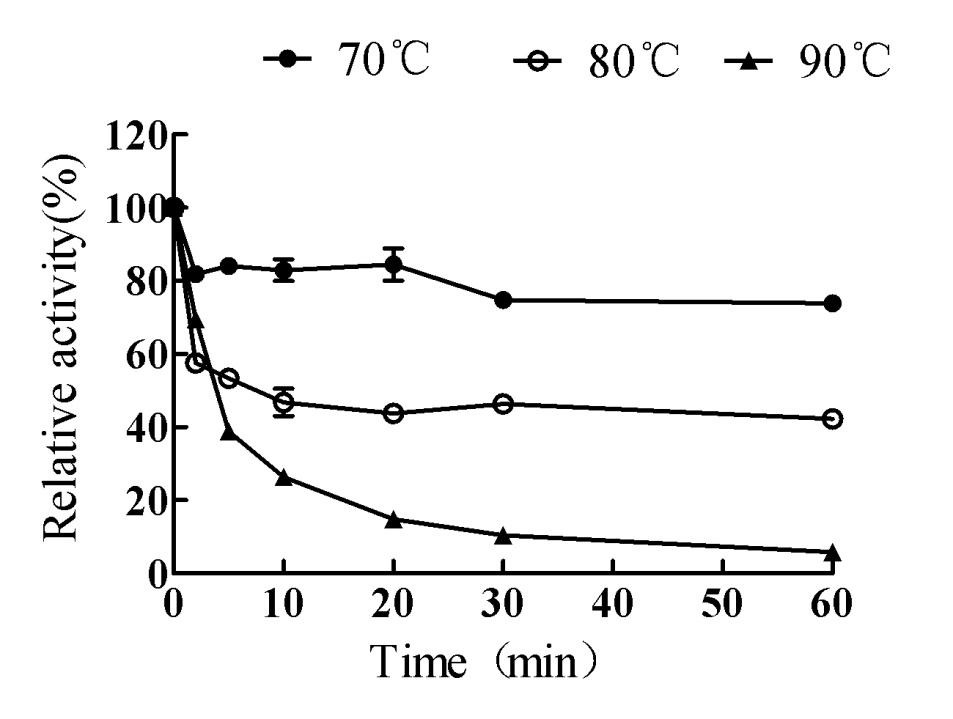


**S2 Fig..Thermostability of the full-length *Cb*Cel9B/Man5A.** *Cb*Cel9B/Man5A was incubated at 70°C, 80°C, and 90°C for 1 h. At different time, samples were taken out and measured for the residual mannanase activity. Locust bean gum was used as the substrate. The activities of *Cb*Cel9B/Man5A were represented as relative activity (in percentage) by dividing the activities against the reference activity, which was the activity before treatment.
