# Supplementary material for: Identification of the C-Terminal GH5 Domain from CbCel9B/Man5A as the First Glycoside Hydrolase with Thermal Activation Property from a Multimodular Bifunctional Enzyme
Source: PLoS One. 2016 Jun 3;11(6):e0156802. doi: 10.1371/journal.pone.0156802 (PMC4892530; doi:10.1371/journal.pone.0156802)
Supplement: S1 Table — (DOCX) [file pone.0156802.s003.docx]

**S1 Table. Effects of metal ions and chemicals on the activity of *Cb*Man5A**

| **Chemicals** | | **Relative activity（%）** |
| --- | --- | --- |
| None | | 100.0 |
| Co^2+^ | | 140.5±1.6 |
| K^+^ | | 133.8±2.6 |
| Na^+^ | | 131.3±1.0 |
| Ni^2+^ | | 126.3±1.7 |
| Mn^2+^ | | 121.5±1.9 |
| Zn^2+^ | | 118.1±2.2 |
| β-Mercaptoethanol | | 114.0±2.3 |
| Cr^3+^ | | 110.0±1.3 |
| Fe^3+^ | | 105.6±1.3 |
| Mg^2+^ | | 104.3±2.5 |
| Ca^2+^ | | 100.8±2.9 |
| Pb^2+^ | | 93.6±2.0 |
| EDTA | 92.1±1.4 | |
| SDS | | 84.6±1.8 |
| Cu^2+^ | | 75.9±0.5 |
| Ag^+^ | | 9.0±3.0 |
